# Functional recovery of patients with moderate and severe traumatic brain injury after extended and standard early rehabilitation

**DOI:** 10.3389/fnhum.2026.1932368

**Published:** 2026-09-24

**Authors:** N. Keleman, N. Pilipović, Z. Sučević, R. Krasnik, D. Dragičević-Cvjetković, S. Palija, D. Bojinović-Rodić

**Affiliations:** 1 Clinical Rehabilitation Service, University Clinical Center of the Republic of Srpska, Banja Luka, Bosnia and Herzegovina; 2 Faculty of Medicine, University of Banja Luka, Banja Luka, Bosnia and Herzegovina; 3 Institute of Physical Medicine, Rehabilitation and Orthopedic Surgery “Dr Miroslav Zotović”, Banja Luka, Bosnia and Herzegovina; 4 Faculty of Medicine, University of Novi Sad, Novi Sad, Serbia; 5 Institute for Child and Youth Health Care of Vojvodina, Novi Sad, Serbia

**Keywords:** adults, early rehabilitation, functional outcome, intensive care unit, traumatic brain injury

## Abstract

**Introduction:**

Traumatic brain injury (TBI) is one of the leading causes of morbidity, disability and mortality in all age groups. Treatment and early rehabilitation are crucial to the overall level of recovery. The objective is to compare the effects of standard and extended early rehabilitation on the functional recovery of patients with moderate and severe traumatic brain injury.

**Material:**

A prospective experimental study comprised 114 patients with moderate and severe TBI, randomly divided into two groups. The patients of the experimental group had kinesitherapy 7 days a week, twice a day for 45 min, and the patients of the control group had kinesitherapy 7 days a week, twice a day for 30 min. The follow-up parameters were: age sex, clinical characteristics, Glasgow Coma Scale (GCS), Functional Independence Measure (FIM), Barthel Index (BI) on admission and discharge and Glasgow Outcome Score (GOS) at discharge.

**Results:**

114 patients were randomly divided into two groups of 57 patients each. Males dominate (75.4%), the average age of the sample was Me = 59.0. There was no statistically significant difference between the groups in gender, age and clinical characteristics, GCS on admission; 59(51.8%) patient had severe, 55 (48.2%) moderate TBI, at discharge: 12 (10.5%) had severe, 11(9.6%) moderate and 91(79.8%) mild TBI. FIM of the experimental group was 18 vs. 70 and of the control group was 18 vs. 50, BI of the experimental group was 0 vs. 61 and of the control group was 0 vs. 32. GOS at discharge: complete recovery in the experimental group 9, in the control group 7, mild deficit 22 vs. 15, severe deficit 22 vs. 28, vegetative state 4 vs. 7. Normalized improvement (ratio of achieved progress to maximum possible progress) was higher in the prolonged early rehabilitation group: for FIM (48% vs. 30%), BI (61% vs. 32%) and slightly less for GCS (100% vs. 92%).

**Conclusion:**

Although no statistically significant difference on functional recovery between the groups was found, however, a difference in percentage progress was found in favor of extended early rehabilitation, but for a final conclusion it is necessary to make a follow-up after inpatient rehabilitation.

## 1 Introduction

Traumatic brain injury (TBI) is one of the leading causes of morbidity, disability and mortality in all age groups. In the world every year more than 50 million people experience a brain injury. Traumatic brain injury is not only the leading cause of death in the under 45 population, but also the leading cause of death in trauma in Europe (Brazinova et al., 2021). Reported incidence and mortality rates from TBI vary among states and regions. The latest data from the US Center for Disease Control and Prevention show that more than 2 million Americans are treated for TBI each year (282.000 hospitalized and 56.000 died) (WHO World Health Organization, 2006). Data from the European Union differ from those from the United States of America (USA). In relation to the number of inhabitants (EU 510 million, USA 321 million), the number of deaths is lower in the EU. This difference can be explained by the high number of firearm-related TBIs in the US (10.5 per 100.000 population per year). The number of hospital admissions is three times higher in the European Union than in the United States (The World Bank, 2017). The frequency of TBI in developed countries is significantly higher in the elderly due to demographic aging, and in medium-developed and underdeveloped countries traffic trauma is the most common cause of TBI (Li et al., 2016; Rubiano et al., 2015). In the year 2022 in the Republic of Srpska, 54% of patients with TBI were over 60 years old (Keleman et al., 2023). Across all age groups, TBI accounts for 30–40% of all injury-related deaths, and by 2030, TBI is predicted to become the most significant cause of disability (2–3 times higher than disability due to Alzheimer’s disease and cerebrovascular disease together). The costs of treating TBI in the USA are 400 billion dollars per year, which represents approximately 0.5% of the entire annual production (Whiteneck et al., 2016).

The Glasgow Coma Scale (GCS), which is used to assess the level of consciousness immediately after a brain injury, is generally accepted for assessing the level of consciousness after TBI. Taking into consideration the total GCS, all brain injuries are divided into mild (GCS ≥ 13), moderate (GCS 9–12) and severe (GCS ≤ 8) (Sternbach, 2000).

Traumatic brain injury leads to various neurological symptoms such as impaired consciousness and motor control, sensory dysfunction, cognitive disorders and breathing disorders. These changes fare accentuated by the changes that occur due to immobility, mechanical ventilation (MV), sedation and relaxation during hospitalization (Abujaber et al., 2020). The central nervous system has the ability to recover and adapt to secondary compensatory mechanisms.

For a long time, there was a generally accepted opinion in the medical circles that a patient with TBI, especially if the patient is on MV, is “too weak and sick” for early rehabilitation (Dubb et al., 2016). Thanks to medical advances, this opinion has changed significantly, as an increasing number of patients survive TBI, but unfortunately with a high rate of disability. Neurological damage and functional impairment can last for years, even for life. Therefore, there has been an increased focus of interest on the effectiveness of early rehabilitation in improving physical, cognitive, psychosocial, and functional impairments after TBI.

Rehabilitation after TBI should be started as early as possible, if there are no contraindications (Lang et al., 2020). There are still no clearly defined guidelines on the intensity, frequency and duration of physical therapy, so they differ from country to country.

The goal of early rehabilitation is to improve motor and functional recovery, while preventing secondary complications (pneumonia, atelectasis, muscle atrophy, pressure ulcers, deep vein thrombosis, contractures). It is recommended that the intensity and duration of early rehabilitation must be individually dosed depending on the patient’s general condition and capabilities. At the same time, the approach to early rehabilitation must be multidisciplinary, with the participation of a specialist in physical medicine and rehabilitation, a physiotherapist, an intensivist-anesthesiologist, a neurosurgeon, and, if necessary, other specialties (Gerber et al., 2021; Rai et al., 2021; Formisano et al., 2017).

The consequences of TBI include long-term physical, cognitive, behavioral, and emotional impairments, as well as difficulties with activities of daily living, community reintegration, work, social life, family functioning, and partner relationships (Finotti et al., 2021). Measuring treatment outcomes in TBI rehabilitation is particularly challenging due to the variety and severity of impairments that remain after hospitalization and the post-acute period. The following scales are most often used: Glasgow Coma Scale—GCS, Glasgow Outcome Scale—GOS, Functional Independence Measure—FIM and Barthel Index (BI) (Gerber et al., 2021).

Numerous studies have proven the benefits of early rehabilitation after traumatic brain injury (van der Naalt et al., 2017; Manda et al., 2023; Qannam et al., 2017; Tipping et al., 2017). Early rehabilitation provides a significantly better functional outcome than delayed rehabilitation. Early rehabilitation is associated with improved outcome, in terms of reduced duration of coma and hospitalization, better cognitive level at discharge, better score on functional scales and greater chances of returning home. There is a positive correlation between the intensity of early rehabilitation and the outcome, and it has been proven to have a positive effect on neuroplasticity (Andelic et al., 2012; Hayashi et al., 2025; Cetin et al., 2024; Jang et al., 2019). The aim of the study is to compare the effects of standard and extended early rehabilitation on the functional recovery of patients with moderate and severe traumatic brain injury.

## 2 Material

After the approval of the Ethics Committee of the University Clinical Center in Banja Luka, the Republic of Srpska, the research was conducted as a prospective experimental study that included patients of both sexes, over 18 years of age with moderate and severe traumatic brain injury (defined as a Glasgow Coma Scale of 3–12 upon admission), who were admitted to the Clinic for Anesthesia and Intensive Care and the Clinic for Neurosurgery of the University Clinical Center of the Republika Srpska in Banja Luka, in the period from 05.09.2022. until 15.12.2023. Following initial care and the stabilization of vital signs within 24–72 h of admission all patients were examined by specialists in physical medicine and rehabilitation. All the patients were divided into two groups, 57 of them in each group. The process of randomizing patients into groups was carried out using computer generated random numbers in Microsoft Excel. Allocation concealment was ensured by the fact that the randomization list was locked by an independent statistician, and insight into the assignment to groups (2 × 30 min or 2 × 45 min of kinesitherapy) was made possible only after the stabilization of the patient and an examination by a specialist in physical medicine and rehabilitation. The first group (experimental) consisted of patients who were involved in early rehabilitation 7 days a week, twice a day, lasting 45 min per session which was carried out continuously from the moment of inclusion until the patient’s discharge from hospitalization. The second group (control) consisted of patients who were involved in standard early rehabilitation, 7 days a week, twice a day, lasting 30 min per session which was also carried out continuously from the moment of inclusion until the patient’s discharge from hospitalization. All patients had an identical kinesitherapy plan, which included positioning, pressure ulcer prevention program, passively and actively assisted exercises to maintain and increase range of motion in all segments of the upper and lower extremities, as well as respiratory kinesitherapy. The evaluation of the patients included the registration of the following data: sociodemographic data: gender, age, mechanism of brain injury (traffic trauma, falls, sport injury, other), clinical characteristic- neurodiagnostics (computed tomography-CT, magnetic resonance imaging-MRI), type of injury (epidural hematoma, subdural hematoma, subarachnoid hemorrhage, intracerebral hemorrhage, cerebral contusion, diffuse axonal injury), combination of two or more brain injury (Yes/No), localization of the injury (right hemisphere, left hemisphere, posterior cranial fossa, brainstem), surgical procedure (Yes/No), associated injuries (locomotor system, chest, abdomen, cervical spine and polytrauma), comorbidities(hypertension, diabetes mellitus, asthma), GCS, BI and FIM on admission and at discharge, and GOS at discharge.

The criteria for the inclusion of patients in the study were: persons of both sexes with TBI verified by computed tomography aged 18 years or older who met the GCS criteria (3–12) on admission and hemodynamically stable for early rehabilitation (heart rate 60–130/min, mean arterial pressure 60–100 mm Hg, respiratory rate 5–40 per minute, peripheral oxygen saturation >88%, if the patient is on mechanical ventilation the proportion of inhaled oxygen (FiO₂) < 60% and positive end-expiratory pressure (PEEP) ≤ 10 cm H2O, artificial airway properly secured). During early rehabilitation, an increase in heart rate by 20 beats per minute and an increase in mean arterial pressure by 20 mm Hg were allowed. The criteria for excluding patients from the study were: persons with traumatic brain injury who were hemodynamically unstable (heart rate >130 per minute, systolic blood pressure <90 or >180 mm Hg, acute myocardial infarction or occurrence of arrhythmias), tachypnea or bradypnea, oxygen saturation <88%, temperature >38 °C. Criteria for stopping early rehabilitation—signs of intolerance: sweating, tachypnea >40 per minute, drop in oxygen saturation below 88%, mean arterial pressure <60 or >120 mm Hg, pulse <60 or >130 per minute and deterioration of neurological status (deterioration of GCS by two points, development of mydriasis and development of focal neurological deficit) (Steiner et al., 2016; Eimer et al., 2021).

The required sample size was calculated using G*Power 3.1 software. An *a priori* power analysis was conducted for a repeated-measures analysis with a within–between interaction, considering two study groups and two measurement occasions. Assuming a medium effect size (*f* = 0.25), a significance level of *α* = 0.05, statistical power of 95%, a correlation of 0.50 between repeated measurements, and a nonsphericity correction of *ε* = 1.00, the minimum required total sample size was 54 participants, corresponding to 27 participants per group. The final sample consisted of 114 participants, with 57 participants in each group, and therefore exceeded the minimum required sample size. Initially, a total of 124 patients met the inclusion criteria and were randomized into the study, with 62 assigned to each group. During the follow-up period, 10 patients passed away due to the severity of their primary injuries (5 in the experimental group and 5 in the control group). These patients were excluded from the analysis, leaving a final sample of 114 participants (57 per group) who completed the entire protocol.

## 3 Statistical analysis

Categorical variables are presented with absolute and relative frequencies, while numerical variables are described with the median and interquartile range (Me, IQR), considering the deviation from the normal distribution. The normality of the distribution was tested using the Shapiro–Wilk test, as well as the Skewness and Kurtosis analysis. A significant deviation from the normal distribution was observed, with the presence of floor effects and ceiling effects for certain scales, which is why non-parametric methods were applied in the further analysis. The Mann–Whitney *U* test was used to evaluate differences between two independent groups, while the Wilcoxon rank test was used to evaluate changes between two time intervals (admission and discharge). To control for the risk of false positives due to multiple comparisons, the *p*-values were adjusted using the False Discovery Rate (FDR) correction according to the Benjamini–Hochberg procedure.

In order to further evaluate the effect of the intervention, a normalized improvement was calculated for each of the applied scales. Normalized improvement is defined as the ratio of the achieved progress and the maximum possible improvement in relation to the initial value, according to the formula: Normalized gain (%) = (value at discharge - value on admission)/(maximum scale value - value at admission) × 100. This approach allows a comparable assessment of progress between subjects with different initial values and reduces the influence of floor and ceiling effects.

Due to the pronounced deviation from the normal distribution of results, the presence of floor and ceiling effects, as well as significant heterogeneity of variance, the application of parametric methods such as analysis of covariance (ANCOVA) and mixed analysis of variance (SPANOVA) was not considered adequate.

Statistical significance is defined at the null hypothesis probability level of *p* ≤ 0.05. Statistical processing and analysis was done in the computer program IBM SPSS Statistics for Windows, Version 27.0. (IBM Corp., Armonk, NY, United States).

## 4 Results

The sample consisted of 114 patients, out of which 57 with extended early rehabilitation (experimental group) and 57 with standard early rehabilitation (control group). The sample was uniform in terms of gender and age structure. The sample was dominated by men (75.4%), while the average age of the total sample was Me = 59.0 (IQR = 37.0), with no differences between groups (*p* > 0.05). (Table 1).

**Table 1:** General characteristics of the respondents.

General characteristics	Patients classified according to early rehabilitation			*p*-value
	[All]	Experimental group	Control group	
	*N* = 114	*N* = 57	*N* = 57	
Sex				1.000^а^
Male	86 (75.4%)	43 (75.4%)	43 (75.4%)	
Female	28 (24.6%)	14 (24.6%)	14 (24.6%)	
Age (years)				0.231^b^
18–30	17 (14.9%)	11 (19.3%)	6 (10.5%)	
31–40	14 (12.3%)	6 (10.5%)	8 (14.0%)	
41–50	11 (9.6%)	3 (5.3%)	8 (14.0%)	
51–60	17 (14.9%)	6 (10.5%)	11 (19.3%)	
61–70	20 (17.5%)	9 (15.8%)	11 (19.3%)	
71–80	15 (13.2%)	9 (15.8%)	6 (10.5%)	
81–90	20 (17.5%)	13 (22.8%)	7 (12.3%)	
Age (years) e(IQR), Min – Max	59.0 (37.0), 18–90	64.0 (43.0), 18–90	58.0 (29.0), 21–86	0.402^c^

^a^Chi-Square test, ^b^Likelyhood Ratio test, ^c^Mann-Whitney *U* test, %, percentage.

The clinical characteristics of the patients were analyzed (Table 2). The most common mechanism of traumatic brain injury was falls (50.9%), followed by traffic trauma (31.6%), while other causes were less common (17.5%), and sports injuries were not recorded. Computed tomography was performed in all patients (100%), while MRI was used in 11.4% of patients. Of the types of injuries, the most common were subdural hematoma (60.5%) and brain contusion (59.6%), as well as subarachnoid hemorrhage (51.8%), while other injuries were less common. Combinations of two or more brain injuries were recorded in 63.2% of subjects. Injuries were most often localized in the right (80.7%) and left hemisphere (78.9%), while injuries to the brainstem (5.3%) and posterior cranial fossa (7%) were significantly less common. Surgery was performed in 35.1%. The most common associated injuries were polytrauma (37.7%) and injuries to the chest (25.4%), followed by injuries to the locomotor system (21.9%), while injuries to the cervical spine (10.5%) and abdomen (5.3%) were less common. Among comorbidities, hypertension was the most common (60.5%), while diabetes and bronchial asthma were present in 18.4% of the subjects each. No statistically significant differences were found between the groups for any of the analyzed variables (*p* > 0.05).

**Table 2:** Clinical characteristics.

Clinical characteristics	All	Experimental group	Control group	*p*-value	FDR corrected *p*-value
	*N* = 114	*N* = 57	*N* = 57		
Mechanism of TBI occurence				0.071	0.874
Traffic traumatism	36 (31.6%)	15 (26.3%)	21 (36,8%)		
Sport injury	0 (0%)	0 (0%)	0 (0%)		
Falls	58 (50.9%)	35 (61.4%)	23 (40,4%)		
Other	20 (17.5%)	7 (12.3%)	13 (22,8%)		
Neurodiagnostics					
Computed Tomography	114 (100%)	57 (100%)	57 (100%)	1.000	1.000
Magnetic Resonance Imaging	13 (11.4%)	6 (10.5%)	7 (12,3%)	0.768	0.929
Type of injury					
Epidural hematoma	15 (13.2%)	8 (14%)	7 (12,3%)	0.782	0.929
Subdural hematoma	69 (60.5%)	34 (59.6%)	35 (61,4%)	0.848	0.929
Subarachnoid hemorrhage	59 (51.8%)	26 (45.6%)	33 (57,9%)	0.190	0.874
Intracerebral hemorrhage	17 (14.9%)	6 (10.5%)	11 (19,3%)	0.189	0.874
Cerebral contusion	68 (59.6%)	33 (57.9%)	35 (61,4%)	0.703	0.929
Diffuse axonal injury	13 (11.4%)	5 (8.8%)	8 (14%)	0.377	0.929
Combination of two or more brain injury				0.120	0.874
Yes	72 (63.2%)	32 (56.1%)	40 (70,2%)		
No	42 (36.8%)	25 (43.9%)	17 (29.8%)		
Localization of the injury					
Right hemisphere	92 (80.7%)	47 (82.5%)	45 (78,9%)	0.635	0.929
Left hemisphere	90 (78.9%)	45 (78.9%)	45 (78,9%)	1.000	1.000
Posterior cranial fossa	8 (7%)	3 (5.3%)	5 (8,8%)	0.463	0.929
Brainstem	6 (5.3%)	2 (3.5%)	4 (7%)	0.402	0.929
Surgical procedure				0.239	0.916
Yes	40 (35.1%)	23 (40.4%)	17 (29,8%)		
No	74 (64.9%)	34 (59.6%)	40 (70,2%)		
Associated injuries					
Locomotor system	25 (21.9%)	14 (24.6%)	11 (19,3%)	0.497	0.929
Cervical spine	12 (10.5%)	5 (8.8%)	7 (12,3%)	0.542	0.929
Chest	29 (25.4%)	15 (26.3%)	14 (24,6%)	0.830	0.929
Abdomen	6 (5.3%)	5 (8.8%)	1 (1,8%)	0.093	0.874
Polytrauma	43 (37.7%)	21 (36.8%)	22 (38,6%)	0.847	0.929
Comorbidities					
Hypertension	69 (60.5%)	33 (57.9%)	36 (63,2%)	0.565	0.929
Diabetes	21 (18.4%)	11 (19.3%)	10 (17,5%)	0.809	0.929
Bronchial asthma	21 (18.4%)	10 (17.5%)	11 (19,3%)	0.809	0.929

The likelihood ratio test was performed; TBI, traumatic brain injury; %, percentage.

Differences in scores on the scales between admission and discharge were examined (Table 3). All analyzed scales showed a statistically significant improvement at discharge compared to admission (*p* < 0.001).

**Table 3:** Descriptive statistics and the difference between the two time intervals on the measured tests for the total sample.

Items and total scores	Min–Max	Me	IQR	M	SD	Sk	Ku	Shapiro–Wilk	*p*-value	Effect size
FIM addmission	10–35	18.00	0.00	18.11	1.80	6.664	72.672	0.146**	<0.001	0.789
FIM discharge	18–126	53.00	57.00	60.42	33.23	0.279	−1.151	0.927**		
BI addmission	0–25	0.00	0.00	0.40	2.88	7.643	59.290	0.124**	<0.001	0.763
BI discharge	0–100	37.50	65.50	44.69	34.58	0.063	−1.472	0.895**		
GCS addmission	3–11	8.00	7.00	6.81	3.59	−0.054	−1.902	0.734**	<0.001	0.870
GCS discharge	5–15	15.00	2.00	13.21	3.06	−1.892	2.283	0.620**		

**p < 0.01. Me, Median; IQR, Inter-quartile range; M, Mean; SD, std. deviation; Wilcoxon Signed Ranks Test was performed. FIM, functional independence measure; BI, Barthel index; GCS, glasgow coma scale.

Within-group analysis showed statistically significant improvements from admission to discharge in both the experimental and control groups for FIM, BI, and GCS scores (all *p* < 0.001). The effect sizes were large for all outcomes in both groups (*r* = 0.76–0.87), indicating substantial functional and neurological improvement during hospitalization (Table 4).

**Table 4:** Intra-group comparison of FIM, BI and GCS scores between admission and discharge.

Scales	Experimental group – admission Me (IQR)	Experimental group – discharge Me (IQR)	*p*-value	Effect size	Control group – admission Me (IQR)	Control group – discharge Me (IQR)	*p*-value	Effect size
FIM	18.00 (0.00)	53.50 (67.00)	<0.001	0.82	18.00 (0.00)	49.00 (61.25)	<0.001	0.77
BI	0.00 (0.00)	49.00 (79.25)	<0.001	0.77	0.00 (0.00)	30.00 (69.25)	<0.001	0.76
GCS	9.00 (7.00)	15.00 (1.25)	<0.001	0.84	3.00 (7.00)	14.00 (4.25)	<0.001	0.87

Me, median; IQR, interquartile range; FIM, functional independence measure; BI, Barthel index; GCS, Glasgow coma scale. Wilcoxon signed-rank test was performed.

On the FIM scale, the median increased from Me = 18.00 on admission to Me = 53.00 at discharge, indicating a marked improvement in functional independence (*p* < 0.001). The distribution of results on admission was highly positively asymmetric (Sk = 6.664) with a pronounced leptokurtic form (Ku = 72.672), that is, a strong grouping of values around lower results. At discharge, the distribution was much more uniform (Sk = 0.279, Ku = −1.151).

A similar pattern was observed with the BI, where the median increased from Me = 0.00 to Me = 37.50 (*p* < 0.001). On admission, the distribution was highly asymmetric (Sk = 7.643) and leptokurtic (Ku = 59.290), which indicates a concentration of results at the lowest values, while at discharge the distribution became more symmetrical (Sk = 0.063) and platykurtic (Ku = −1.472), which indicates more widely scattered results.

For the GCS scale, the median increased from Me = 8.00 on admission to Me = 15.00 at discharge (*p* < 0.001), indicating a significant improvement in level of consciousness. The distribution on admission was approximately symmetrical (Sk = −0.054) and platykurtic (Ku = −1.902), while at discharge negative asymmetry (Sk = −1.892) and moderately expressed leptokurticity (Ku = 2.283) were observed, which indicates the grouping of results toward higher values of the scale.

The results indicate not only a significant improvement, but also a change in the shape of the distribution toward greater heterogeneity and a shift toward more favorable outcomes. The Shapiro–Wilk test indicates that the distributions deviate significantly from normality, and in order to analyze the differences between two repeated measurements, the Wilcoxon test was used.

The differences in the results of the measured tests between the experimental and control groups were examined (Table 5). No statistically significant differences between the groups were found for any of the analyzed variables (all *p* > 0.05). On admission, the values of FIM (Me = 18.00), BI (Me = 0.00) and GCS (Me = 8.00) were uniform between the groups, which indicates a similar initial functional and clinical status of the subjects. At discharge, a tendency for better results was observed in the experimental group compared to the control group: FIM (Me = 70.00 vs. 50.00), BI (Me = 61.00 vs. 32.00) and GCS (Me = 15.00 vs. 14.00), however these differences did not reach statistical significance (*p* > 0.05).

**Table 5:** Measured tests of the experimental and control groups.

Scales	Patient classified according to early rehabilitation			*p*-value	Effect size
	[All]	Experimental group	Control group		
	*N* = 114	*N* = 57	*N* = 57		
FIM admission, Me (IQR)	18.00 (0.00)	18.00 (0.00)	18.00 (0.00)	0.574	0.053
FIM discharge, Me (IQR)	53.00 (57.00)	70.00 (63.00)	50.00 (51.00)	0.115	0.148
BI admission, Me (IQR)	0.00 (0.00)	0.00 (0.00)	0.00 (0.00)	0.547	0.056
BI discharge, Me (IQR)	37.50 (65.50)	61.00 (63.50)	32.00 (65.00)	0.199	0.120
GCS admission, Me (IQR)	8.00 (7.00)	9.00 (7.00)	3.00 (7.00)	0.097	0.155
GCS discharge, Me (IQR)	15.00 (2.00)	15.00 (1.00)	14.00 (2.50)	0.079	0.164
GCS categories on admission, n(%)				0.190	0.123
Severe injury	59 (51,8%)	26 (45.6%)	33 (57.9%)		
Moderate injury	55 (48.2%)	31 (54.4%)	24 (42.1%)		
Mild injury	0 (0%)	0 (0%)	0 (0%)		
GCS categories at discharge, n(%)				0.490	0.112
Severe injury	12 (10,5%)	5 (8.8%)	7 (12.3%)		
Moderate injury	11 (9,6%)	4 (7.0%)	7 (12.3%)		
Mild injury	91 (79,8%)	48 (84.2%)	43 (75.4%)		

Median (Interquartile Range) are showen in table. Mann–Whitney *U* test was performed. FIM, functional independence measure; BI, Barthel index; GCS, Glasgow coma scale.

Table 6 shows the normalized improvement expressed as a percentage of maximal recovery achieved. The normalized improvement was calculated as the ratio of the achieved progress and the maximum possible progress in relation to the baseline value of the scale. The percentage of the achieved maximum recovery was calculated according to the formula:


(discharge−admission)/(scale maximum−admission)x100.

**Table 6:** Comparison of normalized improvement between the experimental and control groups.

Scale	Experimental Me (IQR)	Control Me (IQR)	*p*	Effect size	Improvement (%)
FIM	48.15 (57.41)	29.63 (47.22)	0.113	0.152	E: 48% vs. C: 30%
BI	61.00 (62.00)	32.00 (60.00)	0.203	0.125	E: 61% vs. C: 32%
GCS	100.00 (16.67)	91.67 (33.33)	0.069	0.161	E: 100% vs. C: 92%

FIM, functional independence measure; BI, Barthel index; GCS, Glasgow coma scale. Normalized improvement = ((discharge score − admission score)/(maximum possible score − admission score)) × 100.

A greater normalized improvement is observed in the experimental group compared to the control group for all analyzed scales, especially for FIM (48% vs. 30%) and Barthel Index (61% vs. 32%) and slightly less GCS (100% vs. 92%). However, comparison of normalized improvement between the experimental and control groups using the Mann–Whitney *U* test showed that these differences did not reach statistical significance (all *p* > 0.05), with small effect sizes (*r* = 0.12–0.16). The pronounced ceiling effect and the limited range of GCS measurements resulted in 100% normalized improvement in the experimental group, which does not indicate absolute recovery in treatment, but limited discriminativeness of the scale at higher values.

No statistically significant association was found between age and normalized improvement in FIM (*ρ* = 0.071, *p* = 0.452), BI (*ρ* = 0.116, *p* = 0.220), or GCS (*ρ* = 0.058, *p* = 0.541). These findings indicate that age was not significantly associated with the degree of functional or neurological improvement in the present sample (Table 7).

**Table 7:** Association between age and normalized improvement in functional and neurological outcomes.

Improvement outcome	Spearman’s *ρ*	*p*-value
FIM normalized improvement (%)	0.071	0.452
BI normalized improvement (%)	0.116	0.220
GCS normalized improvement (%)	0.058	0.541

FIM, functional independence measure; BI, Barthel index; GCS, Glasgow coma scale; *ρ* – Spearman’s rank correlation coefficient.

GOS at discharge showed that 15.0% of subjects had full recovery in the experimental group, and 12.3% in the control group, mild deficit 38.6% vs. 26.3%, severe deficit 38.6% vs. 49.1%, and vegetative state 7.0% vs. 12.3%. Although the results of the experimental group are better, they did not reach statistical significance (*p* = 0.375). (Table 8).

**Table 8:** GOS at discharge of the total sample, experimental and control group.

GOS	All	Experimental group	Control group	*p*-value
	*N* = 114	*N* = 57	*N* = 57	
Full recovery	16 (14%)	9 (15.8%)	7 (12.3%)	0.375
Mild deficit	37 (32.5%)	22 (38.6%)	15 (26.3%)	
Heavy deficit	50 (43.9%)	22 (38.6%)	28 (49,1%)	
Vegetative state	11 (9.6%)	4 (7.0%)	7 (12.3%)	

## 5 Discussion

Traumatic brain injuries, due to their consequences on the level of functional disability of patients and their quality of life, have great worldwide socio-medical and economic importance. Our sample consisted of 114 patients with moderate and severe TBI over a one-year period. In our sample, men dominated, almost 3 times more often than women. Most of the epidemiological studies published so far at the world level confirm our result. A study by Maiden et al. (2020) on 540 patients showed that the male gender was more frequent 55.4%. In the study by Heydari et al. (2019) on 306 patients, the male gender was represented in 67.6%. In the Republic of Srpska in 2022, men dominated 70% (Keleman et al., 2025).

Our patients with TBI had an average age of Me = 59.0 years, whereby 54.4% of patients were older than 60 years. In studies that included respondents over 18 years of age, the average age was 58.8 years in Singapore, 54.5 years in Germany, 48 in China and 78 years in Italy (Lui et al., 2020; Schwenkreis et al., 2021; Gao et al., 2020; Orso et al., 2024). Studies that examined the elderly population of patients with TBI showed that the average age in Finland was 80.7 years, and in China 72 years (Posti et al., 2020; Yang et al., 2022).

A total of 57 studies recorded the seven most common mechanisms of injury of TBI: traffic trauma, falls, violence, sports injuries, accidents at home or at work, and suicide or attempted suicide (Brazinova et al., 2021). In our study, falls dominate as the mechanism of TBI occurrence in 50.9% of patients, while traffic trauma was recorded in 31.6% of patients. The picture at the global level confirms these two mechanisms as leading ones with different ratios in various countries. Future research should be focused on the prevention of traffic trauma in the younger population and falls in the older population. The gold standard in the diagnosis of TBI is computed tomography (Lolli et al., 2016). All patients from our study underwent a CT scan according to the protocol. If there is a discrepancy between the clinical condition of the patient and the CT findings, nuclear magnetic resonance is indicated (Sanchez-Molano et al., 2023).

In our study, the incidence of epidural hematoma was 13.2%. Chicote Álvarez et al. (2018) showed in their study that in the last three decades there was an increase in the incidence of epidural hematoma from 1.39 to 9.46%. The same study confirmed that in patients older than 65 years, falls as a cause of epidural hematoma increased from 8.33 to 70%.

Subdural hematoma in the adult population occurs most frequently in geriatric patients and is associated with falls as the dominant mechanism of injury (Ostermann et al., 2018). In our sample, 60.5% of patients had a subdural hematoma.

Traumatic subarachnoid hemorrhage is an unfavorable prognostic factor. In our sample, 51.8% of patients had subarachnoid hemorrhage. A study by Broadwin et al. (2024) on 350 patients showed that deterioration occurred in 5.1% of patients.

In our study, intracerebral hemorrhage was recorded in 14.9% of patients. According to the results of studies of patients with traumatic intracerebral hemorrhage, poor prognostic factors are: age > 60 years, accompanying subdural or subarachnoid hemorrhage, larger volume of the primary lesion, lower resulting GCS, coagulopathy and higher D-dimer level (Wu et al., 2020; Wang et al., 2023).

Brain contusion is the second most common form of TBI, it is more common in men in a ratio of 3:1 (Wilson et al., 2024). In our sample, 59.6% of patients had a brain contusion.

In our sample, 11.4% of patients had diffuse axonal lesions. A study by Javeed et al. (2021) showed that moderate and severe TBI with diffuse axonal lesions have an early mortality rate of 25%, while survivors achieve a satisfactory functional result in almost 50% of cases.

In our study, 63.2% of patients had a combination of several types of brain injuries. In Gutowski et al.’s (2018) study, the incidence of these injuries was 25.74%, and in Marhold et al.’s (2023) study, it was 55.5%. The largest number of our patients had right hemisphere injuries (80.7%), followed by left hemisphere injuries (78.9%). Of the total number of our patients, 35.1% had surgery. In the study by Essen et al. (2023), which included 4,559 patients with TBI, 7% had acute surgery.

Considering the manner of injury, there is very rarely an isolated TBI. These are usually polytraumatized patients with injuries to the locomotor system, cervical spine, chest and abdomen. In the total sample, we had 37.7% of patients with TBI who also had polytrauma. In the study by Riemann et al. (2023), 4,225 patients were included, 164 (4%) patients had simultaneous spinal injuries, namely cervical 63%, thoracic 31%, mortality after 6 months was 11%, and only 36% of patients had a complete recovery. Comorbidities in patients with traumatic brain injury can negatively affect the course of recovery and lead to long-term disability (Hanafy et al., 2021). Patients with more comorbidities were associated with approximately twice the likelihood of dying in the acute phase (Dell et al., 2021).

In our research, we used scales (GCS, FIM, BI and GOS) to assess the degree of coma, recovery and level of functionality, which represent the gold standard and have been widely applied in other studies.

Although it has been in use for almost 50 years, the GCS is irreplaceable in the clinical classification of brain injury severity and as well as a predictor of treatment outcome. (Swaminathan et al., 2024; Kodeeswaran et al., 2017) In the total sample, the median GCS increased from Me = 8.00 on admission to Me = 15.00 at discharge (*p* < 0.001). At discharge, the GCS Me of the experimental group was 15, and of the control group was 14. The Glasgow Coma Scale on admission is a good predictor of recovery and outcome of TBI treatment. Low admission GCS is an independent predictive factor for early interhospital mortality and worse outcome in patients with TPM (Ndoumbe et al., 2018). Numerous authors (Kodeeswaran, Yamagami, Skansaar, Ogolo, Réa-Neto) in their studies emphasized age as one of the factors for a worse outcome measured by GCS (Kodeeswaran et al., 2017; Yamagami et al., 2019; Skaansar et al., 2020; Ogolo and Ibe, 2021; Neto et al., 2023). A prospective multicenter study from Brazil showed that older age, Marschall CT classification, presence of subarachnoid hemorrhage, lower GCS, and papilla abnormality were independent predictors of hospital mortality (Areas et al., 2019). Studies that followed recovery after a longer period (3, 6, and 12 months) showed a better outcome measured by GCS (Sadaka et al., 2018; Oujamaa et al., 2017).

On the FIM scale of the total sample median increased from Me = 18.00 on admission to Me = 53.00 at discharge, which indicates an improvement in functional independence (*p* < 0.001). On admission, the FIM of the experimental group was 18, and at discharge 70.0, and of the control group 18 vs. 50, without statistical significance. The FIM is a standardized questionnaire, the most commonly used measuring instrument of motor and cognitive recovery in patients with TBI. According to the results of the study by Oujamaa et al. (2017), partial motor and cognitive recovery can be expected only after 2–6 weeks. Studies have shown that significant motor and cognitive recovery is expected only after prolonged inpatient rehabilitation (Lu et al., 2018; Appiah Balaji et al., 2023). Greater early neuromotor damages in moderate and severe TBI are a poor prognostic sign, but so far there is no consensus about the time of motor recovery. Studies have shown that most motor recovery is achieved within 6 months after the injury, without significant functional improvement in the second part of the year, 30% of patients cannot walk without help even after 2 years, and 43% of patients have balance problems after 4 years. Motor deficit occurs after TBI, but still little is known about how it develops in the long term and how the initial severity of the injury affects the outcome (Lui, 2021; Patil et al., 2017; Jourdan et al., 2016; Corrigan et al., 2023).

The Barthel index is one of the most commonly used measures in neurological rehabilitation, which measures functional independence in activities of daily living through 10 items (Rollnik et al., 2016). Barthel index of the sample on admission was 0, and at discharge was 37.50 (*p* < 0.001). In the experimental group, BI on admission was 0 and 61 at discharge, and 0 vs. 32 in the control group. According to a study by Zhang et al. (2023) on 1,180 patients whereby poor outcome was verified by Barthel index <60, and predictors of poor outcome were: low Barthel index on admission, advanced age, comorbidities and performans of surgical procedures. In the study by Huang et al. (2023), the BI on admission was 58, and at discharge 88. In addition, the follow-up period of our patients was short, and the improvement of functional independence in activities is expected over time, which is confirmed by studies published so far. In the study by Fan et al. (2020), which included two groups of patients with TBI, standard and more intensive early rehabilitation was carried out. The results did not differ even after 3 months, while after 6 months the BI was statistically significantly higher in the group with more intensive early rehabilitation. In the study by Weber et al. (2016), the BI at discharge was 29.8, and after 6 months 83.3.

Although the observed percentage differences in the improvement of FIM and Barthel index between groups do not reach statistical significance (*p* > 0.05), they have a clear clinical significance because they indicate a positive trend of functional recovery in patients with prolonged kinesitherapy. The absence of statistical significance in this phase can be attributed to the short follow-up time in the acute period and the sample size. For this reason, the normalized metric of improvement can be seen as a clinically relevant indicator of the early phase of recovery, whose full potential and statistical confirmation would probably become visible only through long-term follow-up.

The Glasgow Outcome Scale is a prognostic model for the long-term outcome of patients with TBI (Rubin et al., 2019). In our sample, there was no statistical difference in GOS between the experimental and control group. If GOS is used in the first 6 months after the injury, the result does not show a complete functional outcome for the patient, because with time and rehabilitation, improvements can be achieved that exceed the predicted ones (Kumar et al., 2024). Das et al. (2023), in a study that included 14,552 patients, concluded that GOS is related to the severity of the injury. In the case of moderate TBI, favorable outcomes (GOS 4 and 5) were in 88% of cases, there were 10% of unfavorable outcomes (GOS 2 and 3) and 2% of mortality, and in severe TBI 50 patients had a favorable outcome with 50% of unfavorable outcome and 19% of mortality. Mortality was found in 8.46% of operated patients. A study by Manda et al. (2023) that included 1,233 patients with GSC of 5–12, GOS was followed 3 months after TBI, by telephone and video call. The results showed a significant improvement in GOS values.

## 6 Limitation

This study has its limitations, which are primarily reflected in the small number of respondents, the short follow-up period and the age of the sample. Although greater numerical improvements were observed in the experimental group, the differences between the groups were not statistically significant. One of the possible explanations is the limited statistical power of the study, given the great variability in recovery after brain injury. In addition, the estimated effect sizes were small, making it even more difficult to detect statistically significant differences. Therefore, the absence of statistical significance does not necessarily mean that the intervention has no effect, but rather that the study did not have sufficient sensitivity to detect differences in the observed intensity. In our research, we did not obtain statistical significance between the groups, which could be explained by the sample size, as well as the design of the study itself, because we included a small number of patients in a short time interval from only one health center. The research was not conducted as a multicenter study, which would be useful to examine in future research. For a more detailed analysis, follow-up after 3, 6, 9 months would be necessary, because only after this period and prolonged inpatient rehabilitation a greater motor and cognitive recovery is expected.

## 7 Conclusion

Although no statistically significant difference in functional recovery was found between the groups, a higher percentage of progress was observed in favor of extended early rehabilitation. However, to draw a definitive conclusion, it is necessary to conduct a follow-up assessment after the completion of inpatient rehabilitation.

## Data Availability

The raw data supporting the conclusions of this article will be made available by the authors, without undue reservation.
